# The Atlantic salmon (*Salmo salar*) antimicrobial peptide cathelicidin-2 is a molecular host-associated cue for the salmon louse (*Lepeophtheirus salmonis*)

**DOI:** 10.1038/s41598-018-31885-6

**Published:** 2018-09-13

**Authors:** Gustavo Núñez-Acuña, Cristian Gallardo-Escárate, David M. Fields, Steven Shema, Anne Berit Skiftesvik, Ignacio Ormazábal, Howard I. Browman

**Affiliations:** 1 Laboratory of Biotechnology and Aquatic Genomics, Interdisciplinary Center for Aquaculture Research (INCAR), University of Concepción, Concepción, Chile; 2 Bigelow Laboratory for Ocean Sciences, 60 Bigelow Drive, P.O. Box 380, East Boothbay, Maine, 04544 USA; 3 Institute of Marine Research, Austevoll Research Station, Saugeneset 16, 5392 Storebø, Norway; 4 Complex Systems Group, Department of Physics, University of Concepción, Concepción, Chile

## Abstract

Chemical signals are a key element of host-parasite interactions. In marine ecosystems, obligate ectoparasites, such as sea lice, use chemical cues and other sensory signals to increase the probability of encountering a host and to identify appropriate hosts on which they depend to complete their life cycle. The chemical compounds that underlie host identification by the sea lice are not fully described or characterized. Here, we report a novel compound - the Atlantic salmon (*Salmo salar*) antimicrobial peptide cathelicidin-2 (Cath-2) – that acts as an activation cue for the marine parasitic copepod *Lepeophtheirus salmonis*. *L*. *salmonis* were exposed to 0, 7, 70 and 700 ppb of Cath-2 and neural activity, swimming behaviour and gene expression profiles of animals in response to the peptide were evaluated. The neurophysiological, behavioural and transcriptomic results were consistent: *L*. *salmonis* detects Cath-2 as a water-soluble peptide released from the skin of salmon, triggering chemosensory neural activity associated with altered swimming behaviour of copepodids exposed to the peptide, and chemosensory-related genes were up-regulated in copepodids exposed to the peptide. *L*. *salmonis* are activated by Cath-2, indicating a tight link between this peptide and the salmon louse chemosensory system.

## Introduction

*Lepeophtheirus salmonis* (hereafter referred to as salmon lice) is an ectoparasitic copepod that infests both wild and farmed salmonid fishes (mainly of the generi *Salmo*, *Salvelinus* and *Oncorhynchus)*^1,2^. These parasites reside on the fish and feed on their mucus, tissue and blood, reducing feed conversion efficiency and causing sores and immunosuppression^3^. Salmon lice are a major disease problem in farming of Atlantic salmon (*Salmo salar*), costing the industry millions of USD annually in direct losses to keep parasite loads below prescribed levels^3–5^. They also play a role in the decline of some wild salmonid populations^3,6–11^.

For ectoparasites with free-living life stages, such as *L*. *salmonis*, host-finding is a process that is critical to complete the life cycle^12^. Thus, there has been strong adaptive-evolutionary pressure for this species to develop reliable and sophisticated links to their hosts^13^. Salmon lice use a combination of mechanical, visual and chemical sensory cues to identify and locate potential hosts^14^. However, it has been suggested that chemical signals confer the highest specificity for host recognition^15^. The detection of chemical cues released by the host organism (kairomones) provides an adaptive advantage to the parasite. To detect and respond to host-related compounds, sea lice have evolved sensory systems^15,16^, including chemical receptors found on the antennules located in the first antennae^17^. Antennules are responsive to chemical cues associated with salmon hosts^18,19^. This suggests the presence of chemosensory receptor proteins in these olfactory structures. While the responses of salmon lice to general suites of chemical signals have been investigated^18^, including kairomones^20^, none of these compounds have been linked to the salmon lice chemosensory system at the molecular, neurological and behavioural levels in the same study.

The first description of chemosensory receptor proteins in sea lice species was the identification of *ionotropic receptor* (*IRs*) genes in *Caligus rogercresseyi*, which were responsive to the presence of kairomone-blocking compounds^21,22^. These receptors were also linked to some neurotransmission gene pathways, and had expression patterns related to the presence of antiparasitic xenobiotic drugs^23^. A recent study described a group of new *IRs* genes in the salmon louse, *Lepeophtheirus salmonis*, that are related to olfactory transduction, as demonstrated by *in situ* hybridization and RNA-interference techniques^24^. These *IRs* are the only receptor proteins with known function related to olfaction that have been identified in any marine invertebrate species^25,26^. The role of these receptors in olfaction was predicted from their structural similarity to *IRs* found in insects which have known olfactory function^27^. Despite the diversity of genes associated with the chemosensory system in the sea lice, the suite of chemical cues that influence host encounter rates are incompletely described.

Salmon odour containing unidentified chemical cues triggers altered swimming behaviour (related to host-seeking) in *Lepeophtheirus*. *salmonis*^17,28^, and in *Caligus rogercresseyi*, another species of sea lice that was attracted by the presence of salmon host odour^29^. *L*. *salmonis* also exhibit chemoreceptive activity in the presence of salmon extracts from the mucous in the skin tissue, which contains its odour^18^. However, the specific chemical cues that sea lice detect were not identified in these studies. The first attempt to identify host-derived attractant molecules was from salmon-conditioned seawater^20^. More recently, responses of host salmon to sea lice infection were investigated; annotation of cDNA libraries from specific sites of salmon infection by sea lice showed up-regulation of various peptides and proteins^30^. Peptides related to the antimicrobial peptide (AMP) class of cathelicidins appear to be involved in the sea lice host-recognition system^23^. These AMPs have been tested by *in vitro* assays, producing significant effects on frontal filament formation (which favour sea lice infection) and activation of chemosensory-related genes. However, it has not yet been established that exposure to cathelicidins dissolved in water modulates the neurophysiological responses or swimming behaviour of sea lice.

The objective of this study was to determine whether AMPs related to the cathelicidin family could act as chemical cues that trigger genetic, neurological and behavioural mechanisms that could increase *L*. *salmonis’s* host encounter rate. To achieve this, we (1) applied neurophysiological techniques to evaluate if the parasites can detect AMPs in seawater, (2) observed swimming behaviour to determine if a putative host-seeking response was triggered by exposure to salmon AMPs and (3) undertook transcriptomic evaluation of *L*. *salmonis* genes to evaluate whether there was a chemosensory-related response to the AMP.

## Materials and Methods

### Salmon lice culture

Salmon lice, *Lepeophtheirus salmonis*, were obtained from Atlantic salmon reared at the Institute of Marine Research’s (IMR), Austevoll Research Station, Norway. Between 90–110 gravid female lice were collected from infected salmon. Egg strings were separated from the female using a scalpel and placed in a hatching container (100 cm in diameter fitted with a 100 um sieve on the bottom). The egg chambers were suspended in a running seawater bath (20 L min-1) at 8 °C under a 12 hr light: 12 hr dark photoperiod. Sieves were checked daily for the presence of hatched nauplii. Unhatched egg strings were transferred to a new sieve, which was suspended in the water bath. Sieves containing newly hatched nauplii were labelled with the date to generate cohorts of lice of the same age. Larvae were observed under a microscope to evaluate development until the copepodite stage was reached. Groups of copepodids of the same age were kept separately for further analyses. Cultured lice were used for swimming behaviour, neurophysiological and transcriptomics analyses (Fig. S1).

*Lepeophtheirus salmonis* is a copepod and, since this taxa is not covered by the animal use in research regulations, no ethical approval of these experiments was required.

### Behavioural observations

Swimming behavior was evaluated using a three-dimensional silhouette video photography (SVP) system^14,31^. SVP image sequences were analysed by measuring three behavioural variables: % activity (% of animals responding to the signal), swimming speed (velocity of swimming hops in response to the light stimulus) and latency (the time between the start of the light stimuli and when the animals initiated a hop). About 20 minutes of video (50 hz) was recorded for each trial (StreamPix software v5.0, Norpix Inc., Canada). Frame-by frame analysis (MANTRACK software (JASCO Scientific, Canada) was used to characterize the response of the lice to the light signal. An intermittent light was used as a triggering stimulus in all the behavioural experiments. The light was produced using a 1000 W Xenon arc lamp (Oriel Instruments, USA) with an ON:OFF cycle of 13:47 s. This ON:OFF frequency was used because it produces a strong swimming response from copepodids^14^. Animals were exposed to 20 consecutive ON:OFF cycles. Light intensity (141 microwatts/cm) was recorded at the bottom of the tank using an Ocean Optics Flame spectroradiometer. Data was extracted from the first 10 s after the beginning of the light stimulus (ON) in each of the 20 cycles. The response of the animals to the ON:OFF signal was evaluated for the three control group replicates to determine when the swimming activity was most stable in the consecutive 20 cycles of stimulus. This proved to be after 16 minutes. Therefore, the response to the ON:OFF signal at 16 minutes was used to compare the responses among experimental groups. One-way ANOVA (P-value < 0.05) was used to test for between group differences in the proportion of animals responding to light and swimming velocity, while Tukey’s post-hoc test was used to determine where the statistically significant differences were.

### Selection of candidate antimicrobial peptides

Peptides were obtained as described in a previous study for cathelicidin 1 and 2 (Cath-1 and Cath-2) peptides^23^. In this study and additional peptide (hepcidin; Hep) was also included because it has a similar function to cathelicidin peptides (antimicrobial peptides). Synthesis, purification and molecular weight measurement procedures were the same for these three peptides.

Selection of a compound with putative chemoattractant effect on lice was performed by analysing the swimming behaviours of animals after exposure to the peptides. Groups of 150 healthy copepodids were used in each treatment and in the control. Each group was transferred to a 20 × 20 × 20 cm glass aquarium containing 2 L of filtered seawater at 8 °C. Animals were exposed to one of five treatments before stimulation with light: (1) three classes of salmon antimicrobial peptides (AMPs: Cath-1, Cath-2 and Hep), (2) whole-fish extract (WFX) and (3) 6-methyl-5-hepten-2-one reagent (Sigma-Aldrich, USA). WFX preparation was as described in^18^. The 6-methyl-5-hepten-2-one (6m-5h-2-one) compound is a ketone that was previously identified as a kairomone detected by *L*. *salmonis* during the host-recognition process^20^. Each group of animals was exposed to the treatments for 5 minutes prior to stimulation with the light. A sixth group of animals were placed into an aquarium without any compounds and for 5 minutes before the first stimulus, which was considered as control group. Different glass tanks were used for each experimental group to avoid cross-contamination of compounds, and there were three replicates of every treatment group. Swimming behaviour observations were conducted in order to determine the number of animals that responded to the light stimulus after being incubated with the various test compounds. Selection of the candidate compound for further evaluation was based on the peptide that triggered the strongest responses in the animals (number of copepodids responding and swimming velocity). That compound was Cath-2.

### Neurophysiological evaluation of salmon lice exposed to cathelicidin-2

Electrophysiological measurements and analyses were based on a technique modified from Fields *et al*.^18^. Adult female *L*. *salmonis* were removed from the skin of salmon cultured at the Austevoll Research Station and maintained as described above. Animals were immobilized in a 4 cm diameter petri dish using insect pins (size-000) driven into Syl-Guard (a non-toxic silicon elastomer: Sigma, USA). During the experiment, the antennules were exposed to a constant 1–2 ml/min flow rate of filtered seawater at 8 °C in a movement-damped Faraday cage. Chemical stimuli corresponded to three concentrations of Cath-2 AMP: 7, 70 and 700 ppb, while the control group was filtered seawater. A 200 μm (outer diameter) capillary tube was positioned next to the tip of the antennule to expose distal chemoreceptors to the different concentrations of Cath-2. To record the neural activity of chemoreceptors, the antennule was perforated with 5 MΩ tungsten electrode (FHC, Bowdionham ME, USA) with a 1 μm recording tip. The voltage signal was normalized to a silver reference wire mounted in the water bath and amplified 10X using a DC pre-amplifier and secondarily amplified up to an additional 10000X using an FHC Xcell -3 Microelectrode Amplifier (FHC- Bowdoinham ME, USA). Signals were pre-filtered for 50/60 cycle noise using a HumBug (Questscientific Instruments) and subsequently stored digitally at 96k Hz using commercially available software (Datawave, Fort Collins CO, USA) The data was analyzed off-line using signal processing software (Datawave) and the neural responses sorted based on their waveform characteristics (e.g. peak and valley amplitude, rise time, offset slope) in order to identify individual neurons^32^. We used this analysis to determine instantaneous spike frequency and the number of spikes occurring in response to each stimulus presentation. All responses were corrected for background activity in response to filtered seawater and, where appropriate, control solutions. A one-way ANOVA to test for differences between Cath-2 concentrations and control group.

### Silhouette video photography analyses of swimming behaviour after exposure to different cathelicidin-2 concentrations

Groups of 150 animals of the same age were obtained as described above. Four experimental groups were used to determine the response of *L*. *salmonis* to different concentrations of Cath-2: 0 (control), 7, 70 and 700 ppb. Swimming behaviour of copepodids was assessed using the same methods as described above, but this time reducing the intensity of the light stimulus in order to obtain larger differences in responses that would be more related to the peptide that was added than to the change in light intensity (assuming that less light would produce less activity in copepodids, and the activity increase resulting from the chemical activator would thereby be relatively stronger). The light stimulus was reduced by a factor of 100x using a neutral density filter (ND2), resulting in an intensity of 1.4 microwatts/cm in the aquarium. Data recording, extraction and analyses were performed as described above. All the trials were conducted in triplicate.

After approximately 20 minutes of ON:OFF light stimulus, with the corresponding incubation in Cath-2, all of the experimental groups were filtered using a vacuum suction and filter paper in an Erlenmeyer flask to remove the seawater and all the animals were collected. Copepodids were stored in different cryogenic tubes for each treatment group and fixed in RNA Later solution (Ambion, USA) for transcriptomic sequencing analyses. This was conducted for the three replicates of each group exposed to Cath-2 at different concentrations, including the three control group replicates.

### Tracking the movement of salmon lice after exposure to cathelicidin-2 using the random walk test

A variance ratio test (random walk test) was used to evaluate whether the parasite’s movements are responses to the stimulus or are just random^33^. This test is widely used to study random variations and stochastic patterns^34^.

The swimming paths of all of the individuals from the different experimental groups were evaluated by calculating the displacement of every individual in x, y and z directions. These calculations consisted of measuring the total distance per unit time in one direction: *q*(i + 1) – *q*(i), where “*q*” corresponded to the direction x, y or z. The displacement of any individual was equal to √((distance-x)^2^ + (distance-y)^2^ + (distance-z)), while the total displacement of an individual is the sum of the displacements during each time unit.

Displacement in each direction was tested using the variance ratio test as a time series, comparing every time point with the subsequent point to evaluate if the path was following stochastic behaviour (equations are provided in electronic Supplementary Material 1). Comparisons of this test in the “z” direction indicated presence or absence of stochasticity in vertical swimming (i.e. toward the ON:OFF light stimulus).

### Transcriptomic analyses of salmon lice exposed to cathelicidin-2

RNA extractions of the experimental groups were performed using the Trizol reagent (Invitrogen, USA), following the manufacturer’s protocol. In this case all the 150 copepodids were pooled and RNA was obtained for the controls and for lice exposed to the different concentrations of Cath-2. The isolated RNA was used for whole transcriptome sequencing previously described for sea lice^35^. Briefly, quality of RNAs was measured in a TapeStation 2200 system (Agilent Technologies, USA), concentrations in a QUBIT equip (ThermoScientific, USA). Double-strand cDNA libraries were constructed using the CATS Total RNA-seq kit (Diagenode, Belgium), and were quantified using the NEBNext Library Quant Illumina kit Master Mix for (NewEngland BioLabs, USA). All of the libraries were diluted to 13.5 pM concentration and were sequenced in a MiSeq platform (Illumina, USA), using a 250 × 250 paired-ends reads configuration and 500 sequencing cycles.

Reads obtained from high-throughput sequencing were trimmed by quality and adapters were removed, using the Cutadapt phyton package^36^, using a script detailed by the libraries preparation kit manufacturers. Trimmed reads were used to perform a reference-based assembly using the *L*. *salmonis* genome draft (https://licebase.org/) as a reference in the CLC Genomics Workbench software (version 10.0, Qiagen Bioinformatics, USA) with the default parameters. All the assembled mRNAs from the *L*. *salmonis* genome with coverage higher than 20 in their assemblies were extracted and used as reference for further expression analyses. RNA-seq analyses were performed to measure the expression levels of all of the contigs obtained by the assembly in the four treatment groups using the same software. Expression levels were estimated by calculating Counts Per Millions (CPM), and Kal’s test was applied to calculate fold change values for each Cath-2 concentration over the control group. Contigs having statistically significant differences (fold change > |4|, p value < 0.05) against the control group, or among different concentrations of the peptide, were extracted and annotated using the Gene Ontology criteria (biological processes, molecular function and cellular components) using the Blast2GO software (version 4.0.1, BioBam Bioinformatics S.L., Spain). Direct GO terms counting and GO terms distributions evaluation were conducted using the same software. Three specific gene sets were evaluated: chemosensory receptors, olfactory transduction and synapse. A group of genes of these processes belonging to sea lice species (*L*. *salmonis* and *C*. *rogercresseyi*) were used as a reference, which were obtained from previous studies^21,24^. The gene expression patterns of these genes were measured in all of the experimental groups and statistical analyses were conducted using the same tests and procedures as for the behavioural data.

Transcriptomic data is public in Sequence Read Archive (SRA) of NCBI Genbank database. Accession number: SRP135658.

## Results

### Identifying candidate peptide to test

**Figure 1:**
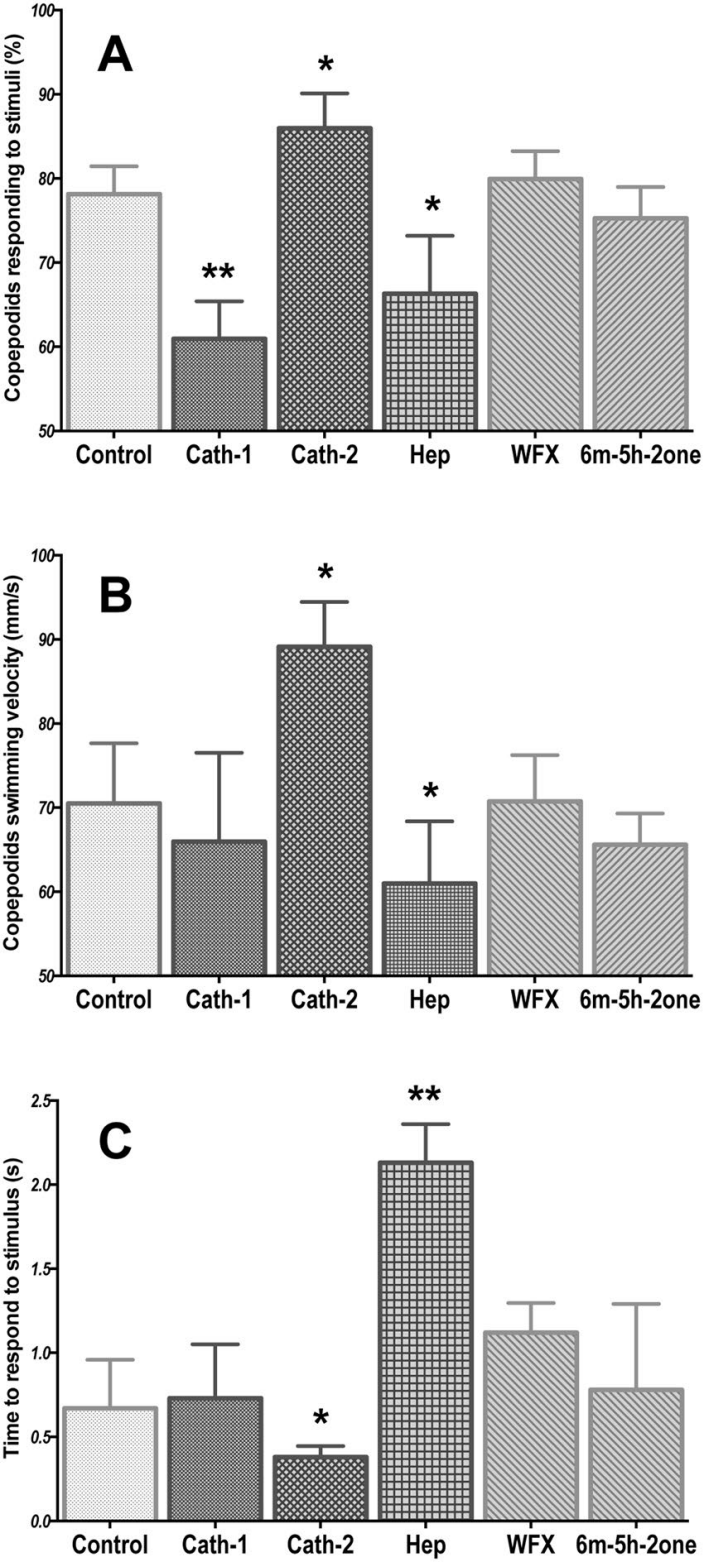
Swimming behavior analyses of *Lepeophtheirus salmonis* copepodids to full light stimulus after incubation with five different compounds. (**A**) Percentage of copepodids that respond to the stimuli; (**B**) mean velocity of copepodids that responded to the light stimulus in each group; (**C**) median of the time that the copepodids spent before responded to the light stimulus. Statistical differences relative to the control group are identified as *p-value < 0.05; **p-value < 0.01. Control: group with no chemical in the seawater, Cath-1: cathelicidin-1 peptide, Cath-2: cathelicidin-2, Hep: hepcidin, WFX: whole-fish extract, 6m-5h-2one: 6-methyl-5-hepten-2-one (N = 150 copepodids per group).

There was a significant effect of the addition of three peptides on the percent of the population responding to the ON:OFF signal. Activity was reduced by 17.19% by Cath-1 (p < 0.01), 11.84% by Hep (p < 0.05), and was increased by 7.83% by Cath-2 (p < 0.05) (Fig. 1A). There was a significant increase in the swimming speed in the presence of Cath-2 (18.63 mm/s faster than control animals, p < 0.05), and a significant speed reduction in the presence of Hep (9.51 mm/s slower than control animals, p < 0.05) (Fig. 1B). There was a significant latency effect caused by Hep (1.46 s more time to initiate the response than control animals, p < 0.01), and also a significantly reduced latency associated with exposure to Cath-2 (Fig. 1C). No significant effect was observed in any of the three variables in the presence of WFX or 6m-5h-2-one compounds. On this basis, Cath-2 was selected for further analysis.

### Neurophysiological response of salmon lice to cathelicidin-2

We recorded chemosensory responses from 7 chemosensory neurons in adult female *L*. *salmonis*. While the ideal life cycle stage to use in this experiment would have been the infective copepodid stage, it was not possible to obtain recordings from them because of their small size. A typical recording contained activity from 1 to 2 identifiable neurons. Not all neurons showed a response to the chemical stimuli. In the 5 animals that responded to the chemical cues the instantaneous spike frequencies to full strength Cath-2 ranged from approximately 10 to 38 spikes s^−1^, with background activity ranging from 0 to 10 spikes s^−1^. We found no neural activity in response to the companion peptide (Cath-1) suggesting that results are not a general response to the peptide but rather specific to Cath-2.

**Figure 2:**
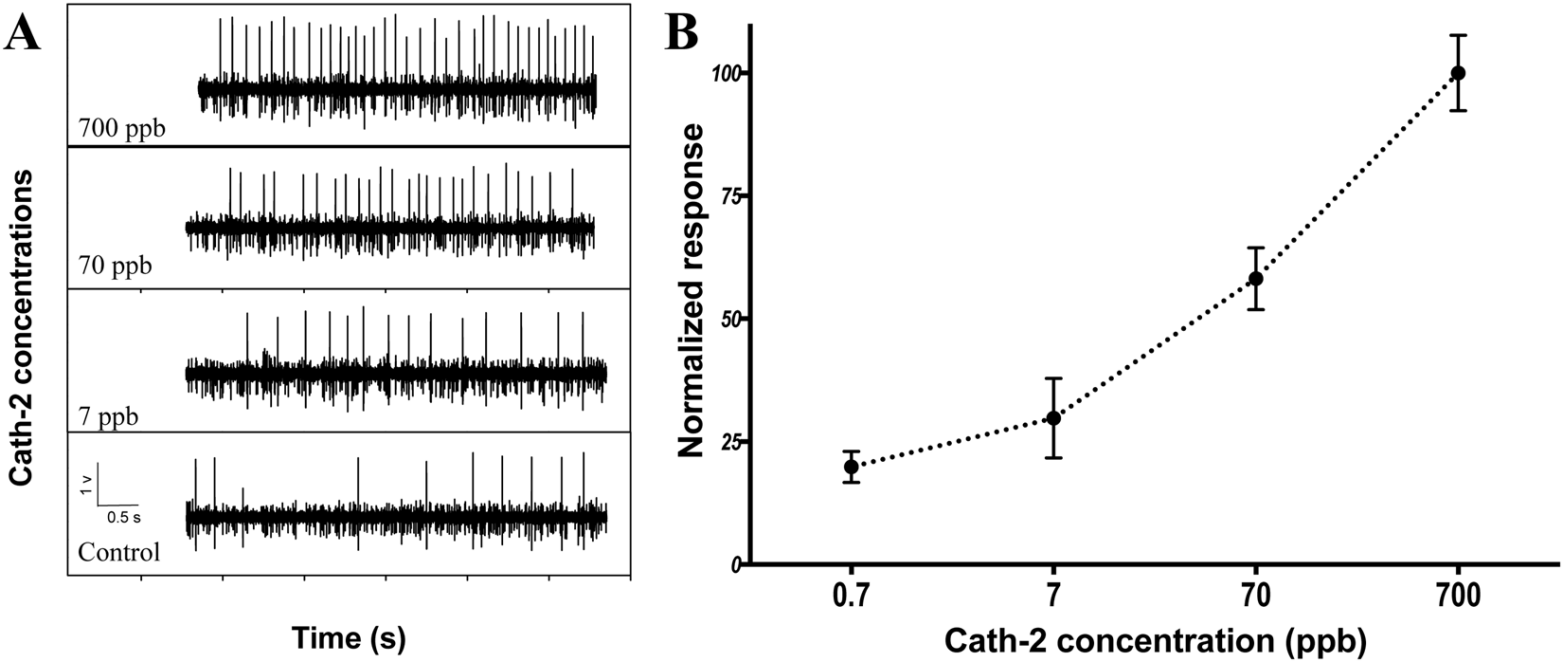
Neurophysiological test of *Lepeophtheirus salmonis* chemoreceptor activity after exposure to different concentrations of cathelicidin-2 peptide (N = 15 adult females). (**A**) Normalized response of neuronal activity (frequency of membrane potential recording) after exposure to the peptide; (**B**) recording of chemoreceptor activity after exposure to cathelicidin-2 at different concentrations. Statistically significant differences relative to the control group were identified at 70 and 700 ppb of cathelicidin-2.

There was a clear electrophysiological response of animals to Cath-2 with higher firing frequency in the neural activity with increased concentration of the peptide (Fig. 2). Stimulation by 700 ppb of the peptide caused the strongest bursts in neuron activity, eliciting a greater response frequency in the chemosensory action potentials than other groups (Fig. 2A). In addition, stimulation with 700 ppm Cath-2 was often accompanied by burst of strong motor activity, in conjunction with visible movement of the antennule or the legs. The neurophysiological response was repeatable within a single louse, showing little evidence of adaptation carried over to subsequent trials. This observation suggested that the 60 s flushing of the chamber was sufficient to remove stimuli from the previous trial. In general, the neural activity for all the lice tested showed a rapid increase in firing frequency in response to an ‘OFF–ON’ chemical signal and on average generated a 10 fold increase in firing frequencies to the highest dose of the peptide. Individual neurons showed a pronounced dose response to increasing concentrations of Cath-2 (Fig. 2B), and typically displayed a log-linear increase in firing rate over the dose range. However, we found no indication that the neural activity saturates at a concentration of Cath-2 above 700 ppb. The pooled data showed a robust log-linear dose-response function over the 3 orders of magnitude increase in concentration of Cath-2. There is no evidence that the animals responded to Cath-1 at concentrations up to 700 ppb. A concentration of 0.7 ppb of Cath-2 showed a similar response as filtered seawater and Cath-1 suggesting it is at the lower limit of detection for the animal.

### Swimming behavior of salmon lice copepodids after exposure to cathelicidin-2

**Figure 3:**
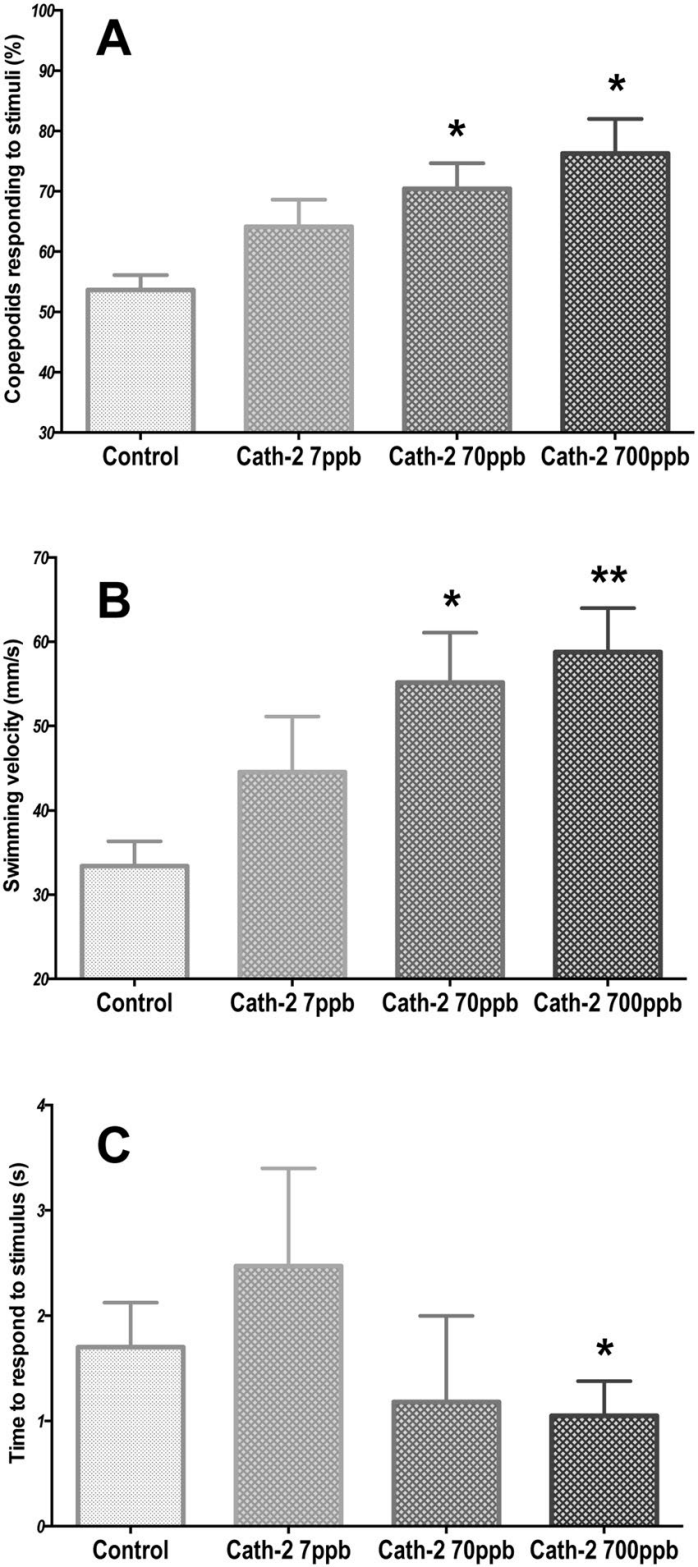
Swimming behavior analysis of *Lepeophtheirus salmonis* copepodids to reduced light stimulus (ND2 filter) after incubation with cathelicidin-2 at three concentrations (N = 150 copepodids per group). (**A**) Percentage of copepodids that respond to the stimulus; (**B**) mean velocity of copepodids that responded to light in each treatment group; (**C**) median of the time that the copepodids spent before responding to the light stimulus. Statistical differences relative to the control group are identified as *p-value < 0.05; **p-value < 0.01. Control: no chemical in the seawater.

The behaviour of the salmon lice was stimulated by the change in light from high intensity (1000 W) to the OFF position after exposure to different concentrations of the Cath-2 peptide. Under this large difference in light intensity, there was a small and non-significant effect of Cath-2 concentration. These results suggest that the behavioural response of the animals may be saturated by the light stimulus, such that exposure to the peptide had little additional effect. However, after reducing the intensity of light signal by 2 orders of magnitude (ND2 filter), the swimming behavior of copepodids increased significantly with Cath-2 concentration (Fig. 3). At the lower light stimulus levels, the number of animals swimming towards the ON:OFF signal changed between control and the higher concentration of the peptide by 25–30%. Significant differences were found for both 70 ppb and 700 ppb of the compound (Fig. 3A). Swimming velocity increased from 32 mm/s (control group) to 60 mm/s (700 ppb Cath-2) (Fig. 3B). Significant changes in velocities were found for 70 ppb (p-value < 0.05) and 700 ppb (p-value < 0.01) compared to controls. In contrast, the latency time to respond to the ND2-light stimulus did not show significant changes with different Cath-2 concentrations, except for animals exposed at 700 ppb of the peptide (Fig. 3C).

### Stochasticity of salmon lice swimming behavior after exposure to cathelicidin-2

**Figure 4:**
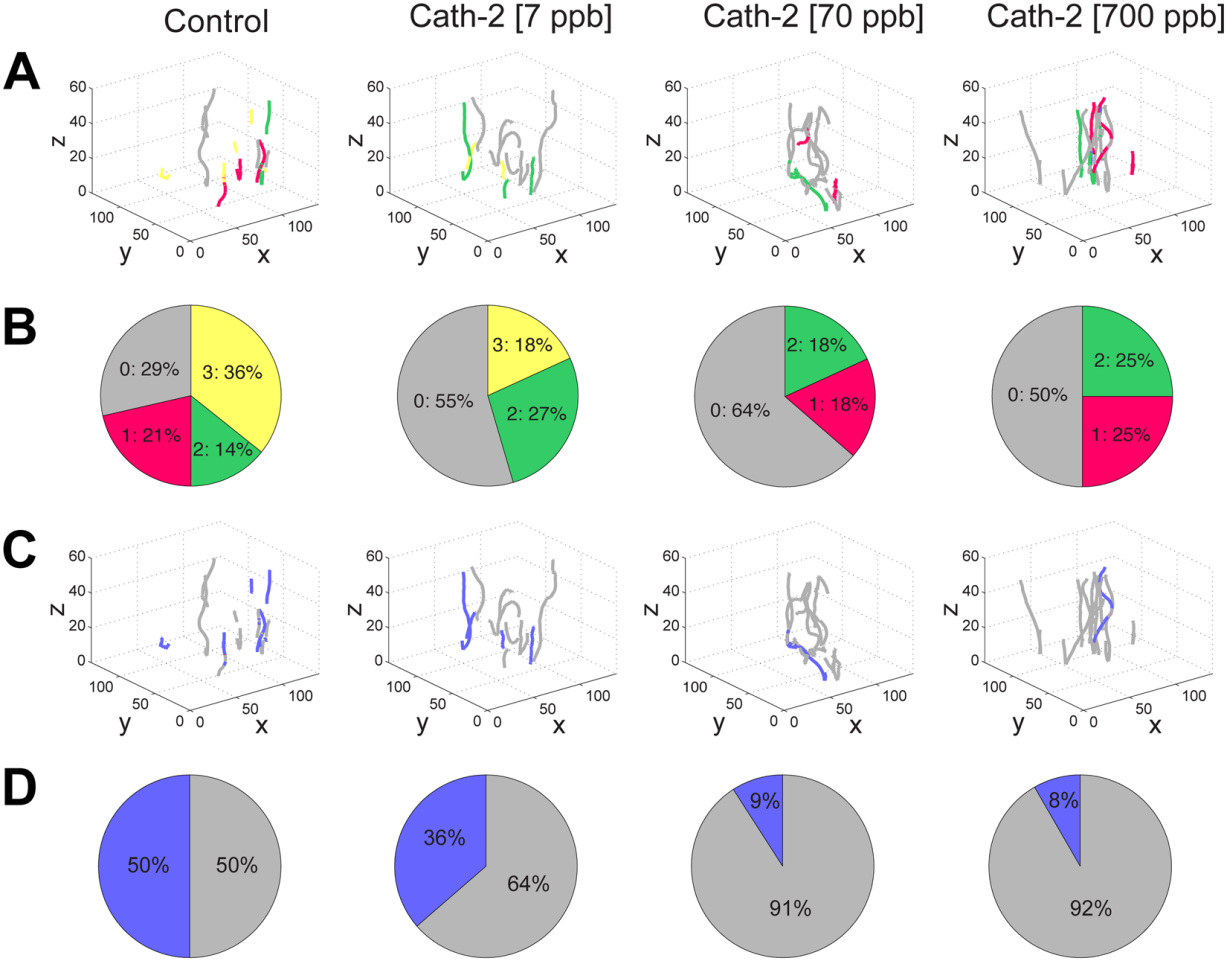
Analyses of trajectories of *Lepeophtheirus salmonis* copepodids during the response to the light stimulus after incubation with three concentrations of cathelicidin-2 (N = 150 copepodids per group). (**A**) 3D-paths of animals responding to the light stimulus and with random walk (stochasticity) in any of the component of the movement trajectory. Grey lines corresponded to paths with no random walk in any component, red lines paths of animals with random walk in one of the three components (x,y or z-axis), green lines animals with random walk in two axes and yellow lines in the three components of the movement simultaneously. (**B**) The distribution of copepodids moving with random walk in 0 (grey), 1 (red), 2 (gray) or 3 (yellow) components. (**C**) 3D-paths of copepodids to the light stimulus and with random walk in the z-axis (vertical movement): grey lines indicate no random walk in the z-axis, and blue indicates that the z-axis component has random walk during the light stimulus. (**D**) Proportion of animals with presence (blue) or absence (grey) of random walk in the z-axis.

There was a significant Cath-2 concentration-dependent reduction of animals exhibiting stochastic movement patterns: 71% of animals had stochastic movement in the control, 45% in those exposed to 7 ppb of Cath-2, 36% for 70 ppb and 50% for 700 ppb (Fig. 4A,B). Considering only vertical swimming, 50% of animals exhibited stochastic paths in the control, 36% in animals exposed to Cath-2 at 7 ppb, 9% at 70 ppb, and 8% at 700 ppb (Fig. 4C,D).

### Transcriptome expression of salmon lice in response to exposure to cathelicidin-2

**Figure 5:**
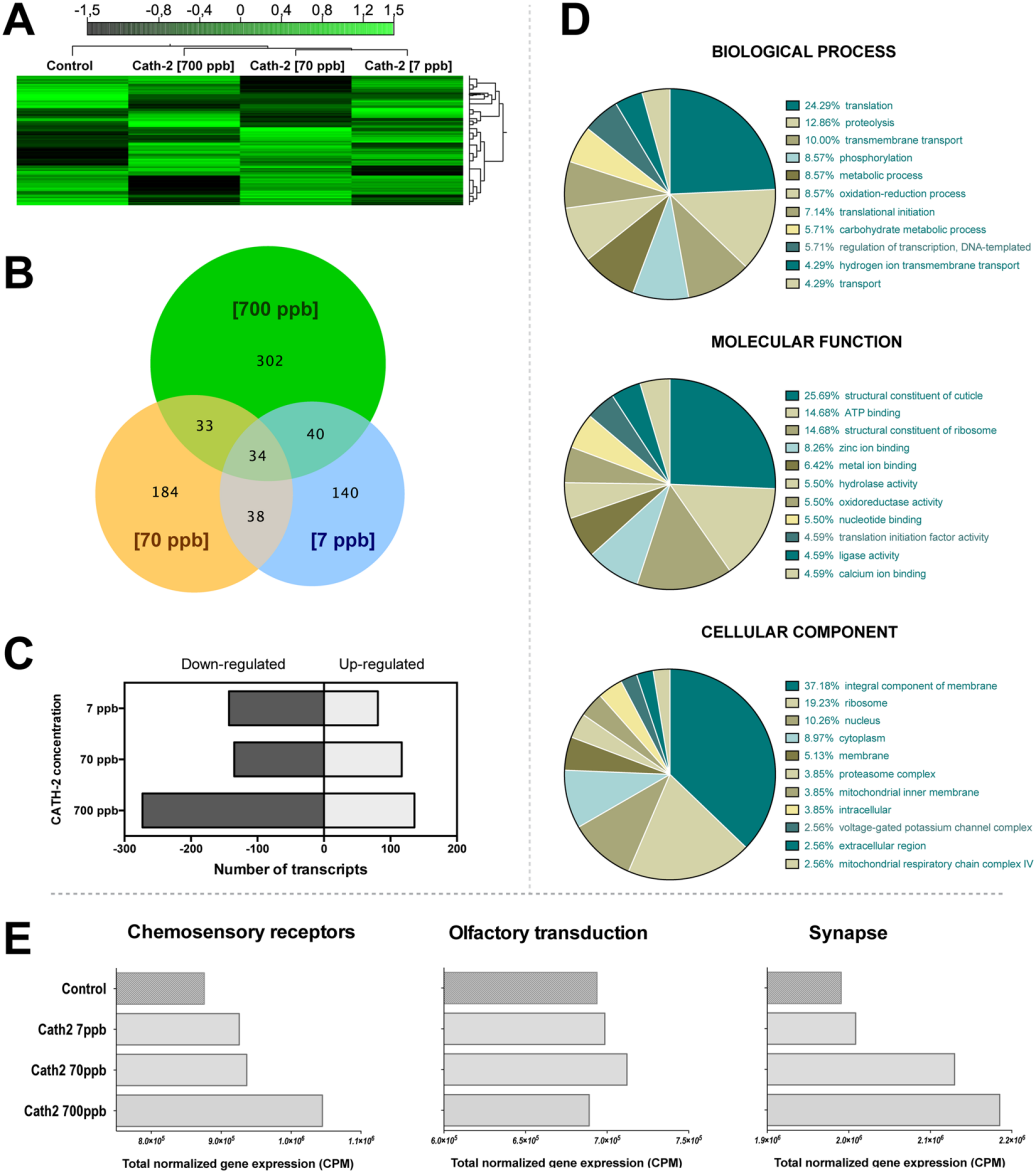
Transcriptomic analyses of *Lepeophtheirus salmonis* copepodids exposed to different concentrations of cathelicidin-2 (N = 150 copepodids per group; three pools for sequencing replicates). (**A**) Heatmap showing hierarchical clustering of gene expression (count per million, CPM values) linked by averages of Manhattan distances according to concentration of the peptide. (**B**,**C**) Number of significantly expressed transcripts (fold change > |4|; p-value < 0.05) in copepodids at each of the concentrations of the peptide against the control group. (**D**) Top-ten most enriched gene ontology (GO) terms according to biological processes, molecular functions and cellular components of significantly expressed transcripts of any concentration of cathelicidin-2 relative to the control group. (**E**) Normalized expression values (CPM) of selected transcripts.

Hierarchical clustering of differentially expressed transcripts exhibited a unique cluster for the control group and another grouping the expression of animals exposed to the peptide at the three concentrations, indicating transcriptome expression patterns that depend on the presence of Cath-2 (Fig. 5A). Statistical comparison revealed that more of these differentially expressed transcripts were related to the exposure to Cath-2 at 700 ppb (409 transcripts with FC > |4|). Exposure to 70 and ppb 7 ppb of Cath-2 produced significant transcription expression in 252 transcripts and 289 transcripts, respectively. Common genes that were significantly expressed in the three conditions corresponded to 34 transcripts, while most of the exclusively expressed transcripts were associated with the 700 ppb exposure, corresponding to 302 transcripts (Fig. 5B). Furthermore, there was a positive relation between the number of up-regulated transcripts and concentration of the peptide. Down-regulated transcripts were also positively related to Cath-2 concentration (Fig. 5C). In total, 1684 transcripts were differentially expressed in the statistical comparisons of gene expression among control and experimental groups.

Annotation (Gene Ontology criteria) of these transcripts revealed that the most important biological processes varying across experimental groups were translation (24% of transcripts), proteolysis (12.86%) and transmembrane transport (10%). A complete annotation list is presented in Supplementary Materials File 2. Regarding molecular functions, the most enriched term was structural constituents of cuticle (25.69%) and ribosome (14.68%), and ATP binding (14.68%). In term of the cellular components, most of the differentially expressed contigs were had positive hits with integral components of membrane (37.18%), the ribosomes (19.23%) and 10.26% in the nucleus (Fig. 5D). Selected transcripts related to chemosensory receptors, olfactory transduction and synapses, also were differentially expressed due to the presence of Cath-2 peptide (Fig. 5E). The chemosensory receptor group included a subset of G-protein coupled receptors (GPCR) that are related to the response to chemical stimuli: non-IGluR ionotropic receptors, variants of metabotropic receptors, NMDA receptors, and chemosensory proteins. This class of membrane receptors were highly up-regulated as Cath-2 peptide concentration increased in the treatments. In contrast, other genes related to olfactory transduction had a different pattern. This subset included all of the genes related to the olfactory transduction pathway but without including the membrane receptors. In this case, the sum of the CPM values did not reveal clear differences. Other genes whose expression was influenced by the Cath-2 treatment, related to the transduction of synaptic signals, including the classic glutamatergic synapse and GABAergic synapse pathway; long-term potentiation and depression; and other synapse transduction pathways, showed a similar trend to the chemosensory receptors.

## Discussion

*Lepeophtheirus salmonis* detect Cath-2 peptide, present at the Atlantic salmon skin and mucous, when it is diluted in seawater. The threshold for the detection of this peptide appears to be between 7 and 70 ppb (Fig. 2). This is a first step towards establishing an ecologically meaningful response threshold for chemical detection of this peptide by free swimming parasites of this species. Further characterization of the threshold sensitivity and response range, and directionality (i.e. repellent or attractant) to Cath-2 will clarify and quantify the role that these cues play in *L*. *salmonis* host-seeking behaviour^37,38^.

In an environment in which there are abundant and diverse chemicals that could act as kairomones or activation cues, signal filtering is critical for optimal detection and chemical communication^39^. The molecular weights of the peptides used in this study were 6.11 kDa for hepcidin, 6.59 for Cath-1 and 5.22 for Cath-2. These weights are in the range of water-soluble compounds detected by *L*. *salmonis* (1 to 10 kDa)^18^. In addition, the approximate concentration of amino acids, among other organic compounds, in the tissues of salmonids ranges from 10^−5^ to 10^−2^ M^40^. These concentrations are consistent with those detected by *L*. *salmonis* based on previous physiological studies (10^−6^ to 10^−8^ M)^18,20^. Combined, these observations support the conclusion that Cath-2 is detected by salmon lice and that it is an activation cue.

Behavioural observation also suggest that Cath-2 will increase the encounter rate of *L*. *salmonis* with its host. Swimming copepodids were stimulated when exposed to the Cath-2 peptide and showed a clear concentration-dependent response (Fig. 3). This is consistent with the neurophysiological results and with earlier studies that reported an increase in swimming speed after chemosensory activation using salmon conditioned sea water^20^. Thus, these results support the role of the Cath-2 peptide as an activator of the chemosensory response in *L*. *salmonis*.

To further assess the effect of Cath-2 on the putative host-seeking behaviour of *L*. *salmonis*, we evaluated the stochasticity level of their swim paths. Copepodids exposed to Cath-2 had lower swim path stochasticity than control groups, suggesting that Cath-2 triggers a swimming response directed towards the light stimuli. When the analysis was restricted to the vertical (z) axis of movement (as has been done for *Daphnia pulex*)^41^, this response was even clearer: most of the stochasticity along the displacement on the z-axis was eliminated when the *L*. *salmonis* copepodids were exposed to Cath-2, especially at higher concentrations. Thus, both electrophysiological and behavioural observations strongly support the interpretation that Cath-2 is an important and potent host-related signal for *L*. *salmonis*. To evaluate whether Cath-2 activated the *L*. *salmonis* chemosensory system at molecular level, transcriptomic expression analyses were conducted.

Exposing *L*. *salmonis* copepodids to Atlantic salmon Cath-2 triggered a broad differential transcriptomic response that was concentration-dependent. This implies that more biological processes were differentially expressed at the highest Cath-2 concentration. This is consistent with the assumption that Cath-2 is a host signal for *L*. *salmonis* – a high concentration would indicate host proximity and the parasite would respond by activating numerous gene pathways. These results are analogous to those reported in other species, especially insects, that exhibit a broad transcriptomic response when exposed to specific chemical cues^42–44^. In aquatic environments, most of the studies on gene expression in response to chemical cues have been conducted on *Daphnia spp*., with results analogous to ours: an up-regulation of various genes^45,46^. Most of the differentially expressed transcripts with GO annotation for molecular function in our study belonged to genes related to the formation of the cuticle structure. This is consistent with a previous study in which copepodids of the sea lice species *Caligus rogercresseyi* were exposed to Cath-2, and cuticle-related genes were up-regulated^23^. These genes are related to moulting events^47^, suggesting that the animals are activating these genes to accelerate their development in response to the detection of a chemical cue indicating that a host is nearby.

According to Gene Ontology criteria of cellular components, most of the differentially expressed genes code for proteins located in the integral component of cellular membranes. This is consistent with gene expression results because chemosensory receptors, such as ionotropic receptors, are located in this membrane^27^. Congruently, a concentration-dependent gene expression level was observed in these chemosensory-related genes, consistent with a previous study in the sea lice species *C*. *rogercresseyi* exposed to the same peptide^23^. We also found an up-regulation of *Ionotropic receptor 25a* gene in a Cath-2 concentration-dependent trend. This gene is one of the most studied olfactory receptor in sea lice species and other invertebrates^48^. On the other hand, synapse-related genes, up-regulated in our experiment also in a Cath-2 concentration-dependent trend, are also relevant as indicators of neurotransmission system in sea lice species^49^. These molecular findings are consistent with the electrophysiological and swimming behavior analyses described above, strengthening the hypothesis that Cath-2 peptide is acting as an activation cue during the host-recognition process of salmon lice.

The strength of the conclusions drawn in this study is based upon the inter-consistency of the observations made at multiple biological levels: physiological, behavioural and molecular. *L*. *salmonis* can detect Cath-2 peptide in sea water through activation of its chemoreceptors, and also respond faster to a stimulus that promotes infestation (faster and more directed swimming), and in parallel, a transcriptomic response related to chemosensory activation and transduction is triggered. These three lines of evidence strongly support the conclusion that the antimicrobial peptide Cath-2, which is produced by salmon, is a molecular fingerprint that activates salmon lice during the host-recognition process.

## Electronic supplementary material

Supplementary information
