# Supplementary material for: The Atlantic salmon (Salmo salar) antimicrobial peptide cathelicidin-2 is a molecular host-associated cue for the salmon louse (Lepeophtheirus salmonis)
Source: Sci Rep. 2018 Sep 13;8:13738. doi: 10.1038/s41598-018-31885-6 (PMC6137231; doi:10.1038/s41598-018-31885-6)
Supplement: Supplementary file 1 — Supplementary information [file 41598_2018_31885_MOESM1_ESM.docx]

**Electronic supplementary materials for**

**The Atlantic salmon (Salmo salar) antimicrobial peptide cathelicidin-2 is a molecular host-associated cue for the salmon louse (*Lepeophtheirus salmonis*)**

Gustavo Núñez-Acuña^1, §^, Cristian Gallardo-Escárate^1, §^, David M. Fields^2^, Steven Shema^3^, Anne Berit Skiftesvik^3^, Ignacio Ormazábal^4^ & Howard I. Browman^3, *^

^1^Laboratory of Biotechnology and Aquatic Genomics, Interdisciplinary Center for Aquaculture Research (INCAR), University of Concepción, Concepción, Chile. gustavonunez@udec.cl; crisgallardo@oceanografia.udec.cl

^2^Bigelow Laboratory for Ocean Sciences, 60 Bigelow Drive, P.O. Box 380, East Boothbay, Maine, USA 04544

^3^Institute of Marine Research, Austevoll Research Station, Saugeneset 16, 5392 Storebø, Norway

^4^Complex Systems Group, Department of Physics, University of Concepción, Concepción, Chile.

Corresponding author:

Howard I. Browman

Institute of Marine Research

Austevoll Research Station

Saugeneset 16

5392 Storebø, Norway

Email: [howard.browman@imr.no](mailto:howard.browman@imr.no)

^§^These authors contributed equally to this work.

**Supplementary text.** Details about random-walk test

The variance ratio test evaluates the null hypothesis of a random walk in a time series of a given variable. It is commonly used in econometric and econophysics analyses to test the hypothesis that the time series, or its first difference (or return, x(t) = y(t) – y(t-1)), is an independent sample and equally distributed or, alternately, that it has hints of random walk.

If “rt” is a random variable, the sum of variances of q values is:


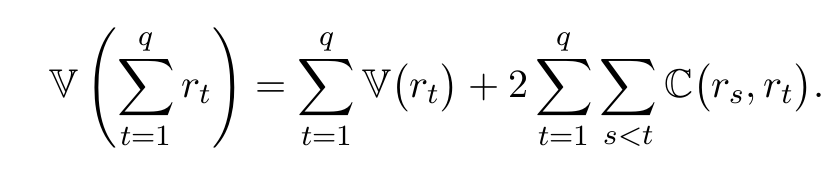


Here, two assumptions were made:

1. Covariance is zero. C(rs,rt) = 0 for “s” distinct than “t”. This helps to define data correlation.
2. Homoscedasticity. V(rt) = (sigma)^2^ * (standard deviation)^2^. This is a property indicating that the stochastic error variances of the data are the same in each observation. This helps to generate predictive models with linear regression and, in this case, helps to predict the subsequent value in the time series.

Therefore:


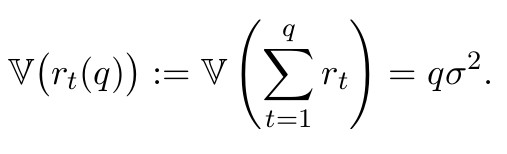


Then, the variance ratio definition is:


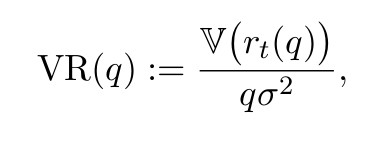


“VR” should be equal to 1 when there is no data according to the time and when there is homoscedasticity.

So, the variance ratio test is a proof:

*H*_0_ : VR(q) – 1 = 0

Versus

*H*_1_ : VR(q) – 1 ≠ 0

If the null hypothesis cannot be rejected, this means that both assumptions are consistent with the data. In contrast, a rejection of *H*_0_ indicates that one of both assumptions are not congruent with the data. Therefore, this test is useful to observe if the data variances are correlated in the time series, and if the associated error is the same in each observation. This on the basis that it is not possible to infer the subsequent observation in a time series with random walking.

In the case if this study, this test makes it possible to infer if the position of copepodids at each time point follows a random walk or exhibits a preference towards a certain stimulus (here, light in the vertical axes) in any direction.


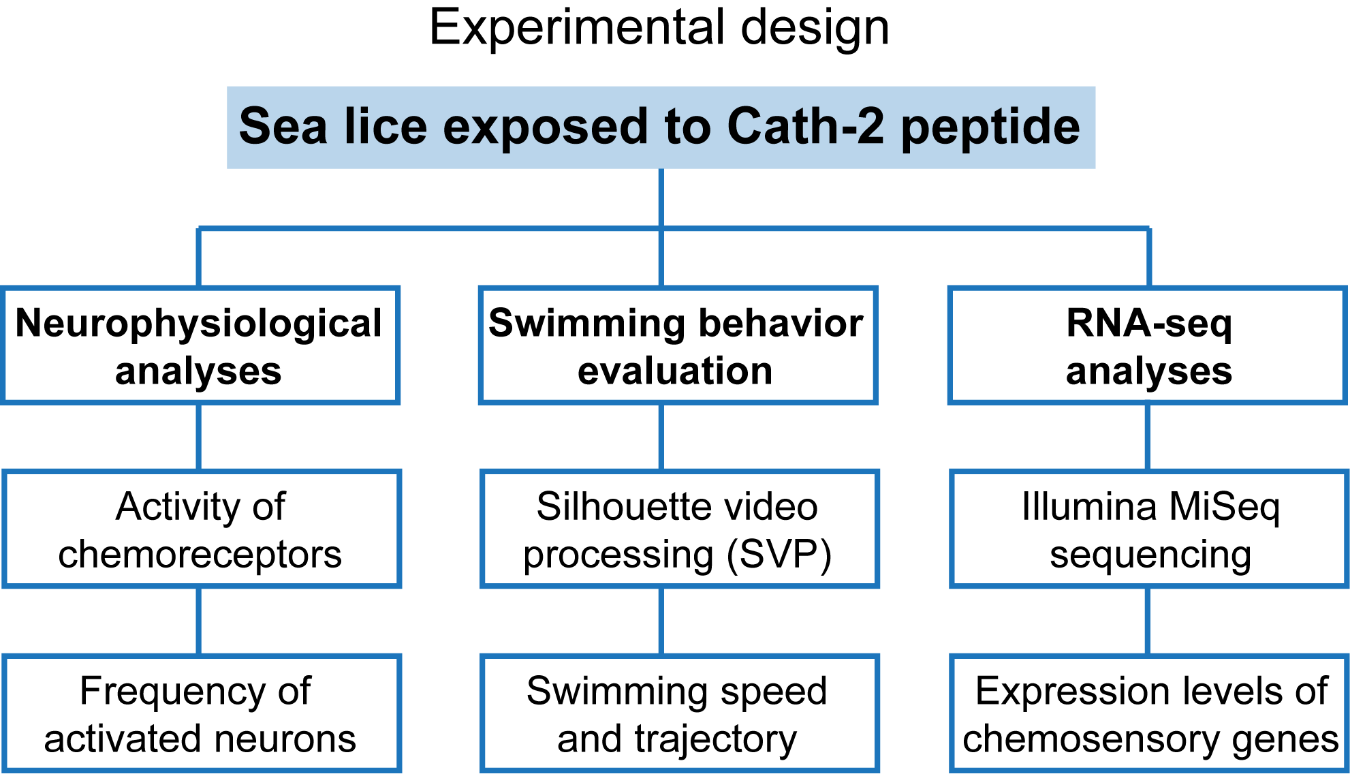


Figure S1. Methodological approach used in this study to infer if the Atlantic salmon (*Salmo salar*) cathelicidin-2 peptide is related to the host-seeking behavior of *Lepeophtheirus salmonis*.


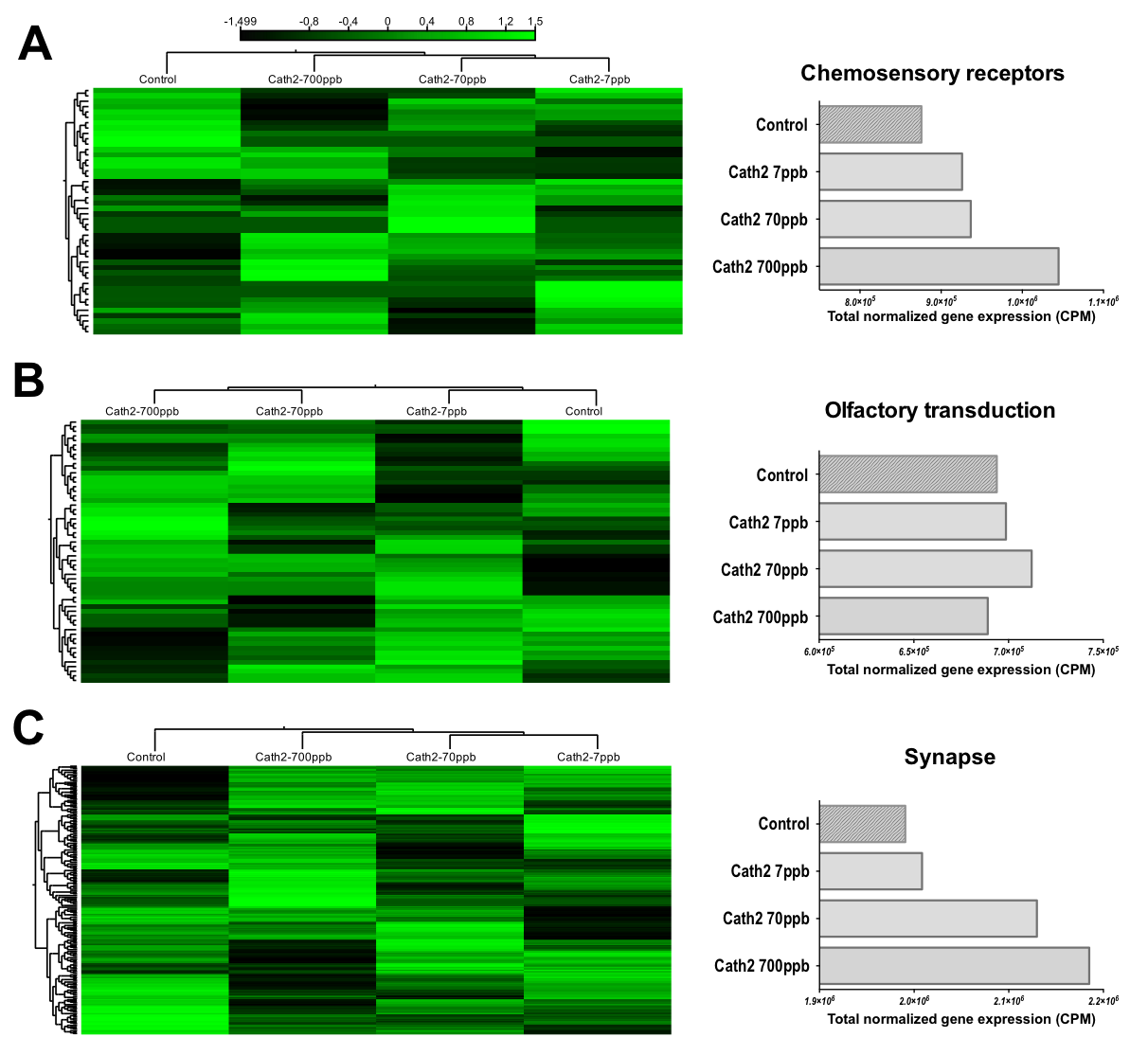


Figure S2. *In silico g*ene expression analysis of selected transcripts of interest in *Lepeophtheirus salmonis* copepodids exposed to cathelicidin-2. On the left, a hierarchical clustering of CPM values of manhattan distances and with average linkage is shown for the four experimental groups. On the right, total normalized expression values (CPM) of the complete set of genes is presented. A: chemosensory receptors, B: olfactory transduction related genes, C: genes related to the transduction of the synaptic signal.
